# Network reconstruction of the mouse secretory pathway applied on CHO cell transcriptome data

**DOI:** 10.1186/s12918-017-0414-4

**Published:** 2017-03-15

**Authors:** Anne Mathilde Lund, Christian Schrøder Kaas, Julian Brandl, Lasse Ebdrup Pedersen, Helene Faustrup Kildegaard, Claus Kristensen, Mikael Rørdam Andersen

**Affiliations:** 1 Department of Biotechnology and Biomedicine, Technical University of Denmark, Søltofts Plads 223, DK-2800 Kgs. Lyngby, Denmark; 2 Recombinant Expression Technologies, Global Research Unit, Novo Nordisk A/S, Novo Nordisk Park, DK-2760 Måløv, Denmark; 3 Novo Nordisk Foundation Center for Biosustainability, Technical University of Denmark, Kemitorvet 220, DK-2800 Kgs. Lyngby, Denmark; 4 Faculty of Health and Medical Sciences, Department of Cellular and Molecular Medicine, University of Copenhagen, Blegdamsvej 3B, DK-2200 København N, Denmark

**Keywords:** Chinese hamster ovary cells, Pathway reconstruction, RNA-Seq, Secretion pathway, Protein secretion

## Abstract

**Background:**

Protein secretion is one of the most important processes in eukaryotes. It is based on a highly complex machinery involving numerous proteins in several cellular compartments. The elucidation of the cell biology of the secretory machinery is of great importance, as it drives protein expression for biopharmaceutical industry, a 140 billion USD global market. However, the complexity of secretory process is difficult to describe using a simple reductionist approach, and therefore a promising avenue is to employ the tools of systems biology.

**Results:**

On the basis of manual curation of the literature on the yeast, human, and mouse secretory pathway, we have compiled a comprehensive catalogue of characterized proteins with functional annotation and their interconnectivity. Thus we have established the most elaborate reconstruction (RECON) of the functional secretion pathway network to date, counting 801 different components in mouse. By employing our mouse RECON to the CHO-K1 genome in a comparative genomic approach, we could reconstruct the protein secretory pathway of CHO cells counting 764 CHO components. This RECON furthermore facilitated the development of three alternative methods to study protein secretion through graphical visualizations of omics data. We have demonstrated the use of these methods to identify potential new and known targets for engineering improved growth and IgG production, as well as the general observation that CHO cells seem to have less strict transcriptional regulation of protein secretion than healthy mouse cells.

**Conclusions:**

The RECON of the secretory pathway represents a strong tool for interpretation of data related to protein secretion as illustrated with transcriptomic data of Chinese Hamster Ovary (CHO) cells, the main platform for mammalian protein production.

**Electronic supplementary material:**

The online version of this article (doi:10.1186/s12918-017-0414-4) contains supplementary material, which is available to authorized users.

## Background

Protein secretion is one of the most important processes in eukaryotes, allowing diverse events from enzyme secretion in saprobes to hormonal signalling in multicellular organisms, and facilitates production of recombinant proteins in most eukaryotic production hosts. Protein secretion is a complex process, which involves a large number of proteins and a series of steps spanning several cellular compartments. The secretory pathway has two main functions: 1) performing proper folding and post-translational modifications (PTMs) of proteins e.g. glycosylation and sulfation, and 2) sorting proteins to their final cellular or extracellular destination. The diverse processes along the secretory pathway are handled by so-called secretory components [30]. The actual protein traffic is regulated by the organised action of numerous structural and regulatory proteins. Additionally, a number of regulatory proteins are dedicated to secure the proper response of the protein secretion pathway to environmental changes, nutrient availability, stress conditions, as well as differentiation signals [24]. In humans, malfunctions in secretory components can result in Huntington’s, Alzheimer’s, or Parkinson’s disease, and protein specific misfolding can lead to cystic fibrosis and antitrypsin deficiency [42, 48].

Such a highly complex process is difficult to describe using a reductionist approach, and therefore a promising avenue is to employ the tools of systems biology. A particularly useful tool is a network reconstruction – a compilation of a list of the known components in a specific area of cell biology and the interaction of said components. Such network reconstructions (RECONs) have helped to analyse complex cellular pathways and networks related to metabolism, transcriptional regulation, protein-protein interactions (PPI), and genetic interactions among others [6]. As RECONs allow the analysis of gene- or protein-level data in their biological context, they become tools for hypothesis-driven biological discovery [34].

To our knowledge, so far none has built dedicated RECONs of the protein secretion pathway with a focus on the secretory components and regulators. Models of the metabolic elements of the secretory pathway have recently been presented for fungi [13, 30]. However, there are few well-defined biochemical reactions in protein secretion, and metabolic models fail to capture all of the regulatory processes and protein interactions. Furthermore, these models have limited applicability in mammalian production systems due to the phylogenetic distance between fungi and mammals. Another approach has been to examine the systems properties of protein secretion through generating a map of the PPIs in the human secretory systems [5, 10]. Such maps provide valuable information about protein organisation and potential protein interactions, but are still static pictures of the interconnectivity. A major weakness of PPI-based networks is that the presence of an interaction between proteins does not necessarily indicate a biologically functional relationship under all conditions [44].

Here, we are interested in applying RECONs to the mammalian secretory pathway and related cell processes due to the importance to biopharmaceutical manufacturing. In 2013, the global market of biopharmaceuticals reached 140 billion USD of which the majority of proteins requiring post-translational modifications are produced in mammalian cells [43].

Among mammalian expression systems, CHO cell-based systems are most commonly used for therapeutic protein production in the biopharmaceutical industry due to the robustness of the cell, their ability to produce glycosylation patterns similar to humans, and that they are well adapted to industrial production in suspension without serum [43]. However, the quality of genome-level data in the CHO system is still at its infancy compared to more developed model organisms such as mouse or humans. The first genome of the CHO cell line was only published in 2011 [16, 46] followed by publications of the draft Chinese hamster genome and several other CHO genomes in 2013 [7, 22, 29]. Therefore, in order to provide a RECON of high quality for understanding protein secretion in CHO cells, one will have to utilize the information from other model organisms, where the annotation is more developed.

In this study, we provide a holistic view of protein secretion which allows the interpretation of genome-scale data from mammalian cell lines, in particular mouse and CHO cells. For this use, we have generated a RECON of the secretory machinery that can integrate data with transcriptomics, proteomics, and genomics. Through manual curation of literature on human and mouse secretory pathways, supplemented by characterizations in yeast, we provide a comprehensive catalogue of characterized secretory components, including with functional annotation and the interconnectivity of the components, thus establishing – to our knowledge – the largest RECON of the functional secretion pathway to date. This serves both as a knowledge repository and as a tool for interpretation of complex genome-scale data from mammalian cells. In this study, we have applied the RECON to transcriptome data from both mouse and Chinese hamster ovary (CHO) cell lines.

## Methods

### Cell culture and media

A suspension and serum-free adapted sub clone of the CHO-K1 parental cell line (ATCC CCL-61), kindly provided by Novo Nordisk A/S, was grown in HyClone CDM4CHO with L-Glutamine medium (Thermo Ficher Scientific) supplemented with 0.5% Penicillin-Streptomycin (Lonza, Thermo Ficher Scientific) and 0.4% Anti-Clumping Agent (Gibco, Life Technologies) (Table 1).

**Table 1:** Chinese hamster ovary cell lines and culture conditions

#	Cell line	Description	Condition
1	CHO-K1	Serum-free/suspension	Control no IgG
2.1	DG44IgG	Serum-free/suspension	Control IgG
2.2	DG44IgG-0NEAA	Serum-free/suspension/0%NEAA	0% NEAA supplement
2.3	DG44IgG	Serum-free/suspension	Secretion stress (NaBu 5 mM)

A recombinant suspension CHO DG44 cell line stabile expressing a human IgG (DG44IgG), kindly provided by Symphogen A/S, was grown in PowerCHO medium (Lonza, Thermo Ficher Scientific) supplemented with 5 mM L-Glutamine (Gibco, Life Technologies), 0.1 mM MEM Non-Essential Amino Acid Solution ((Lonza, Thermo Ficher Scientific), and 0.4% Anti-Clumping Agent (Gibco, Life Technologies). A sub clone of the DG44IgG cell line was adapted to growth without MEM Non-Essential Amino Acid Solution (DG44 IgG-0NEAA) (see Table 1).

All cell lines were expanded in Erlenmeyer cell culture flasks (Corning, Sigma-Aldrich) and grown at 80 rpm in a humidified incubator at 37 °C with 5% CO_2_. Cell viability was measured with NucleoCounter NC-100 cell counter (Chemometec, Allerød, Denmark) according to manufacturers protocol.

### Measurements of metabolites and productivity

Glutamine and glutamate were determined by YSI 2700 Select Biochemistry Analyzer (YSI Life Sciences, USA) calibrated with standard solution from YSI: L-glutamine 2737 and L-glutamate 2756. Glucose and lactate were determined by YSI 2300 Select Biochemistry Analyzer (YSI Life Sciences) calibrated with standard solution from YSI: D-glucose 2356 and L-lactate 1530. The IgG concentration was quantified by Biolayer Interferometry on ForteBIO Octet QK instrument (ForteBIO, USA) using the Protein A biosensor kit according to manufacturer’s protocol.

### RNA purification and next-generation sequencing

Batch cultures were conducted in 250 ml Erlenmeyer cell culture flasks (Corning). The cells were seeded at 3.8 × 10^5^ cells mL^−1^ in 80 ml. The cultures were maintained at 37 °C and a constant agitation speed of 80 rpm. 2 ml were sampled twice a day to monitor the cultures viability and productivity.

In order to analyse the transcriptome, we wanted RNA samples obtained from cells in exponential growth phase as well as in stationary phase. When seeded at 3.8 × 10^5^ cells mL^−1^, CHO-K1 entered the exponential phase within 20 h of cultivation and had not reached stationary phase after 50 h. The CHO DG44 cell lines also entered the exponential phase after 20 h of cultivation and had not entered stationary phase 70 h after seeding.

RNA was extracted from the cultures at the following time points: CHO-K1 at 24 h and after 120 h, DG44IgG and DG44IgG-0NEAA at 48 h and after 120 h, and DG44IgG added sodium butyrate 48 h after inoculation (NaBu, 5 mM) after 140 h (Table 2).

**Table 3:** Mouse RNA-Seq samples downloaded from the Encode Project

Sample name	Tissue	Age [weeks]	Replica #	Sample #	GEO Accession
LID46946	CNS	11.5	1	1	GSM1000573
LID46947	CNS	11.5	2	2	GSM1000573
LID46948	CNS	14	1	3	GSM1000570
LID46949	CNS	14	2	4	GSM1000569
LID46950	CNS	18	1	5	GSM1000570
LID46951	CNS	18	2	6	GSM1000570
LID46983	Placenta	8	1	7	GSM1000565
LID46984	Placenta	8	2	8	GSM1000565
LID46985	Limb	14.5	1	9	GSM1000568
LID46986	Limb	14.5	2	10	GSM1000568
LID46987	Wholebrain	14.5	1	11	GSM1000572
LID46988	Wholebrain	14.5	2	12	GSM1000572
LID47030	Bladder	8	1	13	GSM1000564
LID47031	Bladder	8	2	14	GSM1000564
LID47036	Cerebellum	8	1	15	GSM1000567
LID47037	Cerebellum	8	2	16	GSM1000567
LID47144	Liver	14	1	17	GSM1000574
LID47145	Liver	14	2	18	GSM1000574
LID47146	Liver	14.5	1	19	GSM1000571
LID47147	Liver	14.5	2	20	GSM1000571
LID47148	Liver	18	1	21	GSM1000566
LID47149	Liver	18	2	22	GSM1000566

Total RNA was isolated using phenol–chloroform extraction from Trizol lysed CHO cell pellets. In brief, 2 × 10^6^ CHO suspension cells were washed in ice-cold PBS and lysed in 400 μl TRI reagent (Sigma–Aldrich) and stored at −80 °C. Total RNA was extracted using chloroform and purification was performed by RNeasy mini kit (Qiagen, USA). Concentration and purity were analysed through absorption at 230, 260, and 280 nm using a NanoDrop spectrophotometer (Thermo Scientific) and Qubit 2.0 (Invitrogen, MA, USA). RNA integrity was assessed using RNA 2100 Bioanalyzer (Agilent Technologies, Germany).

Multiplexed cDNA library generation using the TruSeq RNA Sample Preparation Kit v2 (Illumina, Inc., San Diego, CA) and next-generation sequencing were performed by AROS Applied Biotechnology (Aarhus, Denmark) using eight samples per lane in an Illumina Hiseq 2000 system for paired-end sequencing (SRA accession: SRP073484).

### Processing next-generation sequencing data

The FASTQC tool version 0.11.3 (http://www.bioinformatics.bbsrc.ac.uk/projects/fastqc) was used to evaluate the quality of fastq files before and after treatment. Quality trimming and adapter clipping were performed using Prinseq-lite version 0.20.3 [37], trimming trailing bases below quality 20, cutting adaptamer (first 14 bp), and discarding clipped reads shorter than 40 bp. Reads whose mates were discarded due to quality trimming and length constraints were processed as single end reads. The trimmed reads were mapped to the CHO-K1 genome (assembly and annotation) released in 2012 (NCBI Accession: GCF_000223135.1) using TopHat2 version 2.0.9 (using Bowtie 2.2.0) with default settings [25, 26]. Read counts for each transcript were obtained with HTSeq count version 0.5.4p3 using the intersection none-empty mode [1].

In addition to the RNA-Seq data from the eight cultures described above, similar RNA-Seq data of 32 samples from cultures from 14 clones of recombinant suspension CHO DBX11 cell line stably expressing a human factor VIII (FVIII) and RNA-Seq of 22 samples (Table 3) from mouse embryonic tissue were downloaded from the mouse ENCODE project [19, 33]. All RNA-Seq data were processed as described above, but for the mouse RNA-Seq sample reads were mapped mouse genome (mm9, UCSC) (downloaded November 2013, http://ccb.jhu.edu/software/tophat/igenomes.shtml). For each sample only the first 40 million mate-pairs of the 100 million were used.

**Table 4:** The subsystems of the secretory pathway

Subsystem	# Components mouse	# Components CHO cells
Translocation	34	29
Protein folding	103	103
Protein transport	150	138
UPR	65	56
ERAD	128	119

### RNA sequencing data analysis

The read counts were normalised using EdgeR (version 3.6.8) [36] in R [18]. Genes with detected counts per million (CPM) in at least two samples were kept. The normalised read counts were utilised for clustering the major sub-networks gene expression patterns. Hierarchical cluster analysis was performed in R using the package pvclust (version 1.2–2) [40] with average linkage method and the number of bootstrap set to 1000. Main clusters were identified for α = 0.95 and standard errors for approximately unbiased (AU) *p*-values. All genes of the transcriptome dataset were correlated to identify expression profile clusters by calculating Spearman and Spearman squared correlation coefficients. Following identification of the expression levels for all genes in the CHO genome, the Spearman correlation coefficient was calculated for each gene to the productivity of IgG and growth rate μ using R. Genes were considered to correlate significantly with productivity with Spearman’s correlation > 0.81 or < −0.81 (constituting two standard deviations from the mean of all measured correlations).

### Differential gene expression analysis

Differential expression analyses were conducted for the CHO RNA-Seq data of Table 1. To take known sources of variation into account, the differential analyses were performed using the GLM likelihood ratio test in EdgeR for the experiments with multiple factors. A *p*-value of 0.05 and a false discovery rate (FDR) < 0.05 as well as ± log2.0 fold changes, were used as the default thresholds to identify the differentially expressed genes.

### Gene ontology enrichment analysis

A BLASTp search of the CHO proteome from Genbank (downloaded March, 2013) based on the Protein Genbank IDs extracted from the CHO K1 genome annotation file (NCBI Accession: GCF_000223135.1] was performed against the mouse, human and rat proteome from UniProt and The Ensembl BioMart (downloaded March, 2013) to find the closest homologous proteins (lowest *E*-value) in these species. Identifiers, including RefSeq Protein Accession, ENSEMBL gene ID, and UniProt accession for each protein were subsequently obtained using the Gene ID conversion Tool from the DAVID database [17] (from November, 2013). Gene ontology (GO) enrichment analysis was performed by use of the online server of Gene Ontology Consortium [4] and PANTHER classification system [31] using the mouse UniProt accession numbers and *Mus musculus* as background.

### Mouse functional secretory network

A list of components was drafted based on pathway data from mouse retrieved from the Kyoto Encyclopedia of Genes and Genome database [23]. Additional information from UniProt [11] and Reactome [20] of functional annotation and described interaction was included. The draft was refined and expanded by manually curation based on a literature survey of the secretion machinery related genes in yeast, human, and mouse. The genes were categorised in sub-pathways manually according to closest relation found in literature.

### CHO cell line specific secretory network

A local BLASTp of the complete mouse secretion network was performed against the CHO-K1 genome (downloaded from Genbank as assembly GCF_000223135.1 with RefSeq annotation, March 2013). To find the closest homologous of CHO; lowest *E*-value and identity level >90% was considered a CHO homolog.

### Graphic representation of the secretory network

The secretion network was made compatible for visualisation in Cytoscape version 3.2.1 [38]. Colours of nodes were set based on ± 2.0 fold change. Thickness of lines encircling nodes were increased by *p*-value when < 0.05. The significance of the networks is calculated using Fisher’s exact test, and the *p*-value is the executed negative logarithmic transformation.

## Results

### Reconstruction of the mammalian secretory network for mouse proteins

Our first goal was to establish a RECON of the secretory pathway based on the highest possible quality of annotation data. Initially, a draft RECON of the secretory machinery pathways in mouse was generated based on data retrieved from the Kyoto Encyclopedia of Genes and Genome database [23]. Additional information from UniProt [11] and Reactome [20] was included to expand the network beyond the functions covered in KEGG. Furthermore, the draft RECON was curated by adding and refining biological functions found in an extensive literature review of secretion pathway proteins in yeast, human, and mouse. In order to achieve as holistic a view of protein secretion as possible, we also included 75 genes that in literature have been tentatively associated with the secretory machinery. As a result, the generated secretory RECON comprises 801 components, all supported by literature (Additional file 1: Table S1).

Two hundred eighty-seven of the 801 components represent the core components of the protein secretory machinery that are directly involved in the translocation, folding, post-translational modifications and transport of the proteins (Additional file 1: Table S2). The post-translational modifications comprising N- and O- glycosylation systems occurring in the Golgi compartment are seen as independent systems and are therefore not included in this RECON. The reconstructed network thus condenses our current knowledge of the protein secretory machinery excluding the Golgi compartment.

#### Ontology of the RECON: components, subsystems, and functions of the secretory machinery

**Fig. 1:**
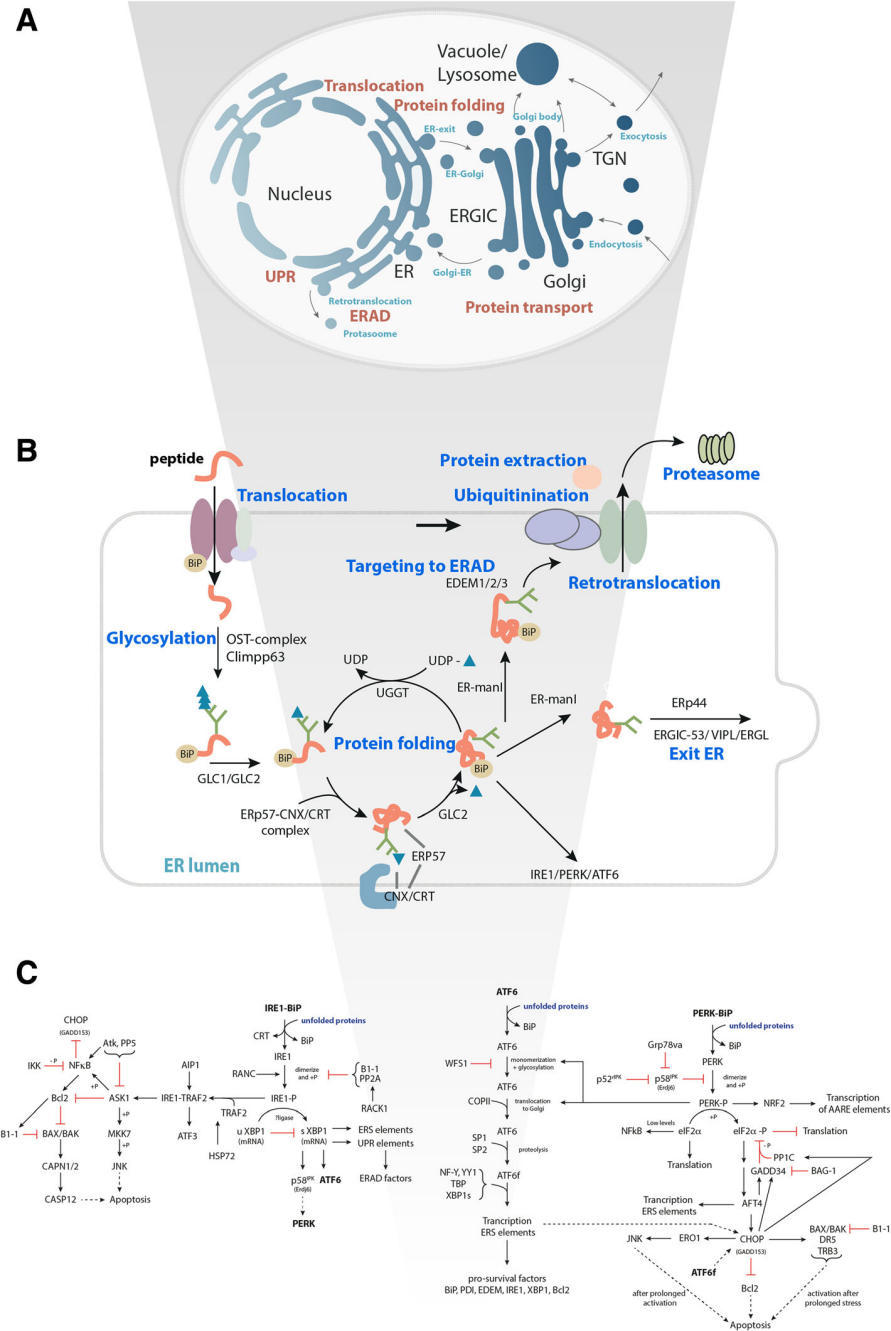
The reconstruction process of the mouse secretory machinery. The process from the overall secretory pathway with: **a** defining the subsystems, **b **classifying functional grouping and protein complexes within the subsystems, to **c﻿﻿ **schematically categorising and adding interactions at the level of sequence, gene, and proteins

The secretory machinery consists of several interconnected pathways defined from literature, which we here termed subsystems. These subsystems are to some extent overlapping: translocation, protein folding (PF), protein transport (PT), ER-associated degradation (ERAD), and unfolded protein response (UPR), see Fig. 1a and Table 4.

**Table 2:** Overview of culture condition at RNA sampling

#	Cell line	Condition^a^	μ [h^−1^]	t_d_ [h]	q_IgG_	Time [h]
1.1	CHO-K1	Exponential phase	0.0353	19.6	0	24
1.2	CHO-K1	Stationary phase	0.001	693.1	0	120
2.1.1	CHO DG44 IgG	Exponential phase	0.0231	30.0	9.93	48
2.1.2	CHO DG44 IgG	Stationary phase	0.0008	866.4	8.28	140
2.2.1	CHO DG44 IgG	Exponential phase, 0% NEAA	0.0235	29.5	8.46	48
2.2.2	CHO DG44 IgG	Stationary phase, 0% NEAA	0.0094	73.7	8.42	130
2.3.1	CHO DG44 IgG	Secretory stress by NaBu	0	−99.0	11.86	70
2.3.2	CHO DG44 IgG	Secretory stress by NaBu	0	−138.6	17.12	100

^a^Condition at the time point of RNA sampling, μ: Specific growth rate, t_d_: Doubling time, q_IgG_: Specific IgG production rate, Time: Time of cultivation

To provide an overview of the 801 components, we first categorized them by the different subsystems. Within each subsystem, components and complexes of components were grouped according to their function described in literature, termed functional groups. A component can be assigned to one or several functional groups if literature reports different functions. A functional network is thus the network of reported interactions within a functional group.

The network was then further expanded by including the following: 1) Branches into the subsystems of autophagy, apoptosis, and ER stress. These branches serve to identify if expression or activity is shifted into these subsystems, which are not as such a part of the protein secretion pathway. Therefore, these branches appear incomplete in term of components. 2) All reports of links between the components, be it DNA-DNA, protein-DNA, or protein-protein interactions (Fig. 1c.)

#### Conversion of the secretory network to a Cytoscape representation for data analysis and visualisation

The complete network of the RECON was made compatible with Cytoscape [38] allowing the integration of omics data for analysis and visualisation. Components with previously described interconnectivity, functional annotation and/or protein complexes were included. As this leaves out components with no described interactions, the Cytoscape representation includes 655 connected components of the secretory RECON. The architecture of the network was expanded to include 42 nodes, which mark protein complexes, as well as the 103 functional groups. Additional nodes were included if isoproteins had previously been reported. The network is provided as a Cytoscape Input File (Additional file 2: Cytoscape input file). Supplying the RECON as a network facilitates extraction of sub-networks for further analysis and the addition of new components and interactions. Furthermore, it serves to ease data interpretation, in general to focus on the part of an ‘omics-dataset involved in protein secretion and in particular to identify co-regulated genes of the same protein complexes and/or from the same function.

#### Test of the functional secretory machinery network for data interpretation

We wanted to test that the reconstruction can be used for data analysis and interpretation. As a first step, we wanted to examine how the the defined subsystems of the secretory RECON represented data, and assess the inference of these systems. In order to achieve this, and furthermore demonstrate the use of the network in relation to transcriptome data analysis, we used an RNA-Seq dataset obtained from the ENCODE project: an assortment of 22 samples of mouse tissue from seven different embryonic tissues of mouse, covering several stages of embryo development [19, 33].

**Fig. 2:**
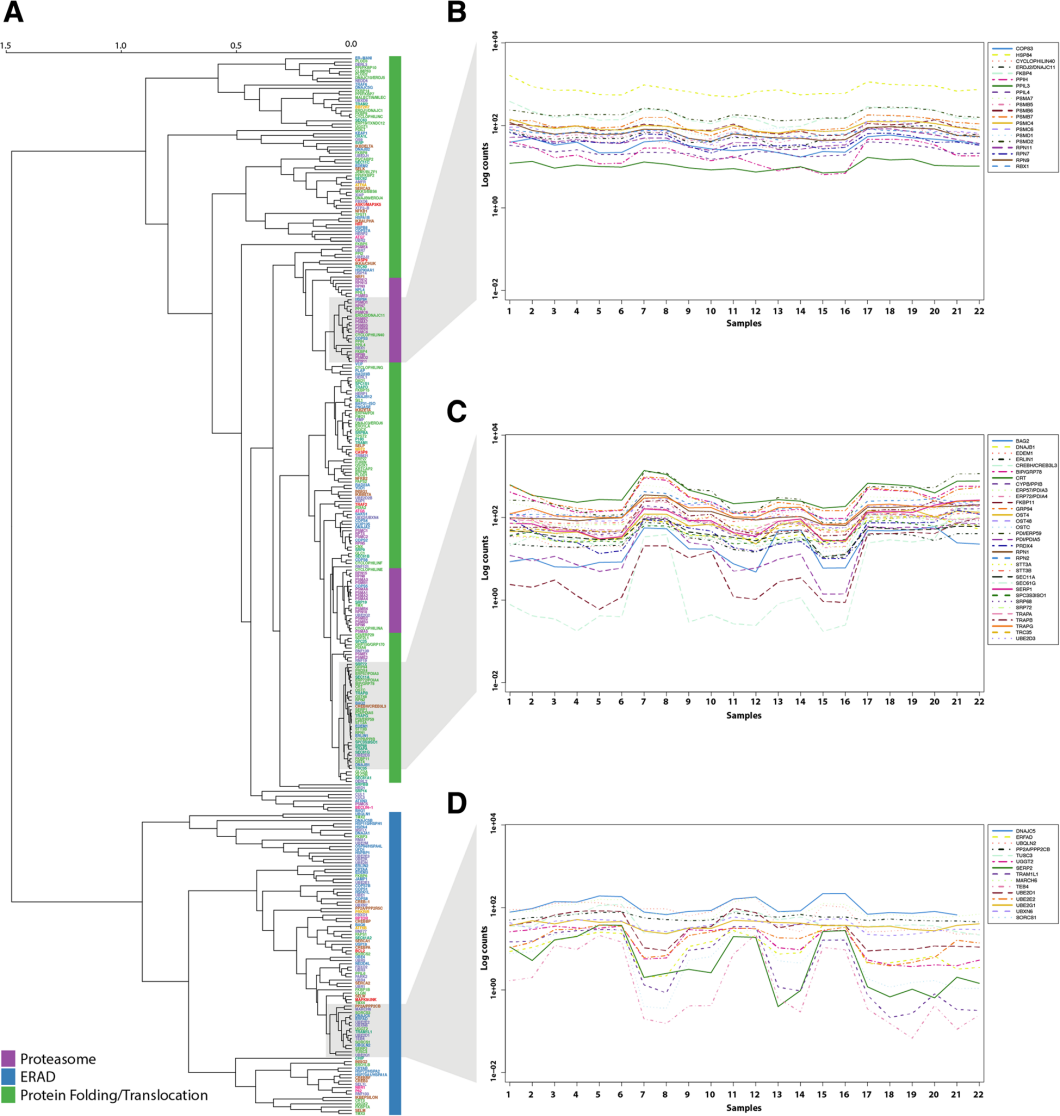
Hierarchical cluster analysis with average-linkage of mouse expression levels. **a** Dendrogram representing the hierarchical clustering of the gene expression levels of the components from the subsystems of ERAD, PF, proteasome, and translocation. Vertical colour bar: *Purple*, Proteasome; *Blue*, ER associated degradation (ERAD); *Green*, Protein folding (PF) and translocation. **b** Gene expression levels across all samples within proteasome. **c** Gene expression levels across all samples within genes with the functional annotation protein folding. **d** Gene expression levels across all samples within genes related to the ERAD. *Blue*, ER associated degradation (ERAD); *Green*, Protein folding (PF) and translocation; *Purple*, Proteasome. 1: CNS_e11.5-1, 2: CNS_e11.5-2, 3: CNS_e14-1, 4: CNS_e14-2, 5: CNS_e18-1, 6: CNS_e18-2, 7: Placenta_8w-1, 8: Placenta_8w-2, 9: Limb_e14.5-1, 10: Limb_e14.5-2, 11: Wholebrain_e14.5-1, 12: Wholebrain_e14.5-2, 13: Bladder_8w-1, 14: Bladder_8w-2, 15: Cerebellum_8w-1, 16: Cerebellum_8w-2, 17: Liver_e14-1, 18: Liver_e14-2, 19: Liver_e14.5-1, 20: Liver_e14.5-2, 21: Liver_e18-1, 22: Liver_e18-2

We performed a hierarchical cluster analysis using RNA-Seq data, as we would expect that strongly co-regulated functional groups and protein complexes will cluster [41]. For each subsystem the gene expression levels of the individual components were extracted and clustered using Spearman correlation to identify monotonic relationships. The clustering was performed with bootstrapping to evaluate stability of the generated clusters. The dendrogram of Fig. 2 shows the average-linkage clustering of normalised count data for 325 components covering the subsystems ERAD, protein folding, and translocation (Fig. 2). Each component is coloured according to a functional annotation allowing evaluation of clustering performance. Functional clusters with the subsystems of ERAD (blue), protein folding (green) and translocation (green) are identified by the vertical colour bar (Fig. 2a). The proteasome that is considered a part of the subsystem ERAD, is highlighted separately due to the large size of this protein complex. Figure 2b-d shows a closer view of the expression patterns across all mouse samples within three separate areas of the clustering.

Our results show here that the clustering of the data (the biological co-regulation, as shown by the dendrogram in Fig. 2) is in very good accordance with the functional categories in the subsystems and protein complexes defined in the secretory RECON (As seen by the colors in Fig. 2).

#### Exploring the potential of clustering according to functions

The RNA-Seq data clustering of the mouse tissue samples allow us to identify new genes potentially associated with the secretory system, based on similar expression profiles suggesting co-regulation [41]. In particular, if a transcription factor is self-regulated, one would expect it to cluster together with the genes it is regulating.

**Fig. 3:**
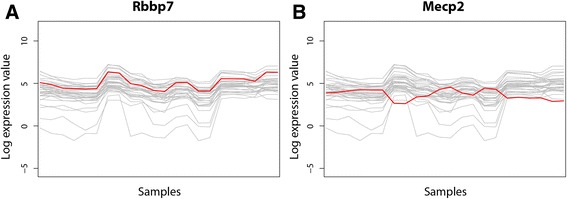
Spearman correlated expression profiles. Expression profiles correlated by Spearman correlation coefficient to the selected protein folding components from Fig. 2c. **a** Expression profile of correlated gene Rbbp7 (*red*) across all mouse samples visualised with the expression profiles of Fig. 2c. **b** Expression profile across all mouse samples of the gene Mecp2 (*red*) correlated by squared Spearman coefficient visualised with the expression profiles of Fig. 2c

To illustrate this potential of the network, we examined the expression profiles of the sub-cluster with the functional annotation ‘protein folding’. The sub-cluster consists of 33 components and is indicated in Fig. 2c. The expression levels of the protein folding sub-cluster was correlated to all individual genes in the complete mouse transcriptome data set and the results were ranked using the summed Spearman correlation coefficients of the individual gene pairs (Additional file 1: Table S4). With this, we identified four genes which are highly correlated to the expression levels of protein folding genes and potentially involved in regulation: Morc4 (a zinc finger protein), Snd1 (a transcriptional co-activator), EIF4ebp1 (a translation initiation factor), and Rbbp7 (a histone-binding protein). In a similar fashion, we identified five genes which have inverse correlation to the protein folding sub-: Tbc1d9, Dock3, Atp6v1g2, Rab3a (all genes involved in signal transduction) and Mecp2 (a methyl-CpG-binding protein). As Fig. 3 shows with the examples of Rbbp7 and Mecp2, the expression profiles are highly similar (or inverse) to the protein folding genes. Thus both proteins could be potential regulators within protein folding.

#### Reconstruction of CHO cells secretory machinery network

The next step was thus to employ the mouse-based secretory RECON to reconstruct the protein secretory pathway of CHO cells using a comparative genomic approach. Through homolog protein search, 726 CHO-K1 genes were mapped to the mouse secretory components with identity over 80% (at the protein level). For an additional 38 ORFs homologs, the identity was only > 60%, although being the best hit, with a significant *e*-value and bit scores above 50 [35]. These proteins were also added to the CHO cell network. Of the identified homologs, 39 were noted partial in the description and two of those components were found to also have partial annotation (SRP54 and CREP). 39 components were not identified by BLAST or annotated as pseudo genes and thus not included.

As a result, the CHO-K1 secretory RECON comprises 764 components (see Additional file 1: Table S1). 270 core components of the protein secretory machinery were identified and the distribution within the major subsystems are listed in Table 3. The graphical representation of the CHO-K1 secretory RECON was created using Cytoscape as described for the mouse network (Fig. 6a).

### Examination of protein secretion in CHO cells

To illustrate the application of the secretory network for analysis of omics data within protein secretion in a cell factory, the secretory RECON was applied to a RNA-Seq dataset from the biopharmaceutical workhorse CHO cells:

A RNA-Seq data set was generated from CHO cells using the following conditions: two growth conditions (exponential growth and stationary phase), two cell lines (CHO-K1 and CHO DG44), with and without expression of IgG antibodies, with and without sodium butyrate (NaBu) treatment, and absence and presence of NEAA in the growth medium (See Table 2). These diverse conditions provide a range of transcript expression levels for genes that are relevant for optimisation of the secretory pathway for heterologous gene expression, with NaBu in particular added to induce secretory stress [12, 39]. The RNA-Seq dataset is experimentally designed to minimise noise from differences between batches and biological variation. Each sample represent a combination of conditions, and the full set secures biological replicates for each condition.

For quality control of the biological replicates, the RNA-seq data was investigated by multi-dimensional scaling (Additional file 3: Figure S1). As expected, the differences between the two cell lines CHO-K1 and CHO DG44 are separated in the first dimension, while the second dimension separates the normal non-treated cells from the sodium butyrate treated cells. The paired nature of the samples, exponential and stationary phases, was confirmed, with the exception of samples with and without NEAA, which seemed to have no effect.

#### Differential expression analyses

As an initial analysis of the data, we identified differentially expressed genes within the four categories: Effects of IgG production, cultivation phases, NEAA medium supplement and secretion stress induced by NaBu (see Table 5).

**Table 5:** Summary of differential gene expression analysis (see Additional file 1: Tables S5–S8)

Condition	Not expressed	Total # analysed	# FDR < 0.05	# |log2 FC| ≥ 2	Up	Down	Disp	BCV
IgG production	8583	16,446	6540	1953	1542	411	0.0168	0.1296
Cultivation phases	8583	16,446	4223	333	265	68	0.0168	0.1296
Secretion stress NaBu	8132	16,897	8121	2857	2316	541	0.01497	0.1224
0% NEAA sup.	9202	15,827	27	5	4	1	0.00588	0.0767

We determined the transcriptional effect of heterologous IgG production in CHO cells by comparing CHO-K1 not producing heterologous proteins (Table 5, 1.1-1.2) with CHO DG44 producing recombinant IgG at industrial levels (Table 5, 2.1.1–2.2.2), using the cultivation phases as blocking. Of the 25,029 examined genes, 16,446 were above the cutoff for expression, and 6540 genes were differentially expressed (false discovery rate (FDR) < 0.05). We identified 1953 genes with |log2 Ratio| ≥ 2, where 1542 were up-regulated and 411 were down-regulated. In a similar fashion, the exponential growth phase was compared to stationary phase with the same set of samples (Table 5, 1.1–1.2, 2.1.1–2.2.2), but using cell lines as blocking. Similar strategies were used to examine the effect of NaBu and NEAA medium supplements (Table 5). In any of the four conditions, the number of genes not expressed was just above 8000. A comparison revealed that these 8000 genes are largely the same in all conditions.

An alternative to the use of our secretory pathway RECON, is the use of the functional annotations from the gene ontology (GO). We thus applied a GO enrichment analysis for comparison to our method: For the CHO-K1 genome, only a limited number of genes have assigned GO-terms. Consequently, we performed a BLASTP search to retrieve mouse UniProt accession numbers that matched the CHO-K1 genome. GO-terms were assigned to the mouse identifiers through the online server of Gene Ontology Consortium [4]. Of the 6540 genes found to be significantly differentially expressed in the IgG production comparison, 1447 genes were mapped to GO-terms using a BLAST comparison of mouse and CHO-K1. A GO enrichment analysis was performed using a cutoff of *p*-value < 0.05 to identify significantly overrepresented GO-terms for each of the main GO categories, biological processes (BP), cellular compartments (CC), and metabolic function (MF) as well as a GO-slim for BP (Additional file 1: Table S9). In summary, the majority of the overrepresented GO terms for BP are regulation or positive regulation of signal transduction (GO:0009966; GO:0009967), signalling (GO:0023056, GO:0023051), response to stimulus (GO:0048584; GO:0048583; GO:0050896), and metabolic, biological or cellular processes (GO:0044710; GO:0048518; GO:0008150). In comparison, the GO-slim enrichment for BP revealed terms that could be associated with the effect of protein production: vesicle-mediated transport (GO:0016192), protein transport (GO:0015031), and intracellular protein transport (GO:0006886). GO enrichment thus gives a broad overview of the cellular processes engaged, but not at the level of detail in the secretory RECON (Fig. 2).

#### Comparative cluster analysis of gene expression levels within the secretory machinery for CHO cells to mouse

**Fig. 4:**
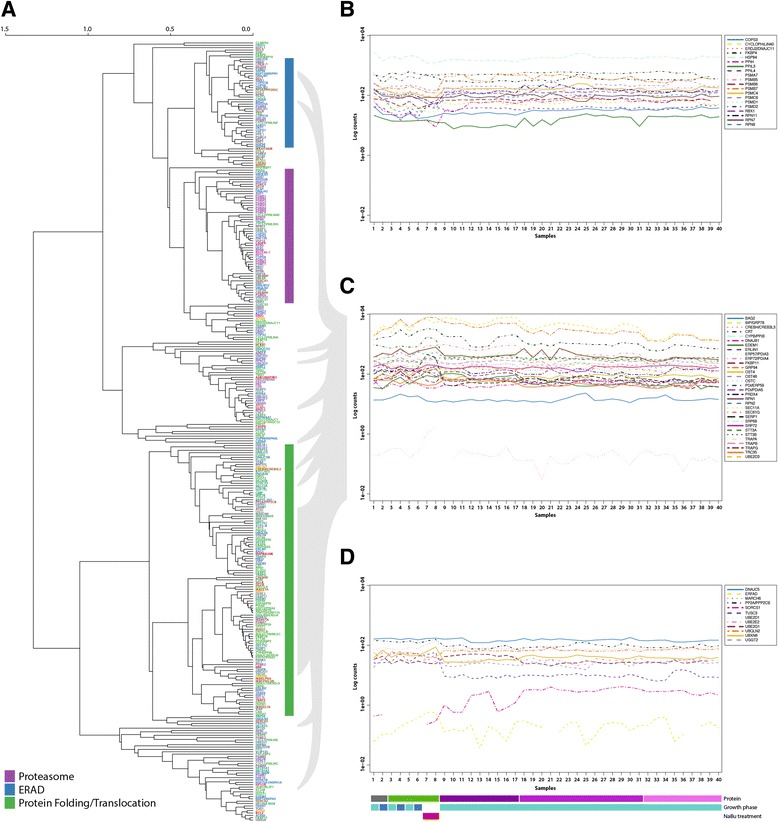
Hierarchical cluster analysis with average-linkage of CHO cells expression levels. **a** Dendrogram representing the hierarchical clustering of the gene expression levels of the components from the subsystems of ERAD, protein folding, and translocation and the proteasome protein complex. Vertical colour bar: *Purple*, Proteasome; *Blue*, ER associated degradation (ERAD); *Green*, protein folding, and translocation. **b** Gene expression levels across all samples for components with the functional annotation proteasome clustering in mouse. **c** Gene expression levels across all samples for components with the functional annotation protein folding in mouse. Grey shadow indicates the position of the components in the hierarchical clustering of the CHO genes. **d** Gene expression levels across all samples for components with the functional annotation ERAD that clustered in mouse. Horizontal bar, identifier of samples. Top line: protein expressed; no recombinant proteins (*grey*), IgG (*green*), and FVIII high levels (*dark purple*), FVIII medium levels (*purple*), FVIII low levels (*light purple*). Middle line: cultivation phase; exponential growth (*light blue*), stationary phase (*Dark blue*). Bottom line: NaBu treatment (*red*)

With the purpose of characterising the expression pattern within the secretion pathway of CHO cells, we applied the secretory pathway RECON for CHO cells to a diverse CHO gene expression dataset. This dataset was composed of 40 different CHO RNA-Seq expression experiments: the 8 samples listed in Table 2, and 32 additional samples obtained from cultivating 14 clones of recombinant suspension CHO DBX11 cell line stably expressing a human factor VIII (DBX11 FVIII) at high, medium and low levels [21]. Hierarchical cluster analysis was performed using Spearman correlation (as described above), for the components related to the subsystems ERAD, protein folding, and translocation, and the proteasome complex. Of these, 303 components are expressed in our data set. The dendrogram of Fig. 4a present the average-linkage clustering of these components. We were still able to identify groups from the subsets ERAD, protein folding, and translocation and the proteasome, but only a part of the genes within a given subset are clustered (Fig. 4a). The expression of the components shown to cluster in mouse (Fig. 2c) are visualised across all CHO samples in Fig. 4b-d. The protein folding group identified in the mouse data is still detectable in CHO cells, but several components cluster differently in CHO cells, as shown in grey in Fig. 4c. Details on the clustering of components of the proteasome and the ERAD are found in Additional file 3: Figure S2. In summary, it seems like functions associated with protein folding are regulated relatively tightly in CHO, but generally regulation is less strict in the CHO cancer cell lines than in the mouse tissues.

Following the changed regulation, we examined the expression patterns of the potential regulators found in mouse expression data (described above). For the gene Mecp2 no sequence homolog could be found in the CHO-K1 genome. The expression of Rbbp7 was plotted against all CHO samples (Additional file 3: Figure S3), but no correlation seems to be present in this data, further supporting the observation of decreased regulation.

#### Gene expression level correlated with protein and growth within the secretory network

We furthermore developed a method to use the genes of the RECON to analyse gene-phenotype correlations for protein production and growth in the secretory network. Extracting all expression values for the 764 CHO genes in the secretory RECON and comparing these with growth rates and IgG titers, we analysed Spearman and Pearson correlations to find monotonic and linear relationships, respectively. Correlation coefficients are available in Additional file 1: Table S10.

**Fig. 5:**
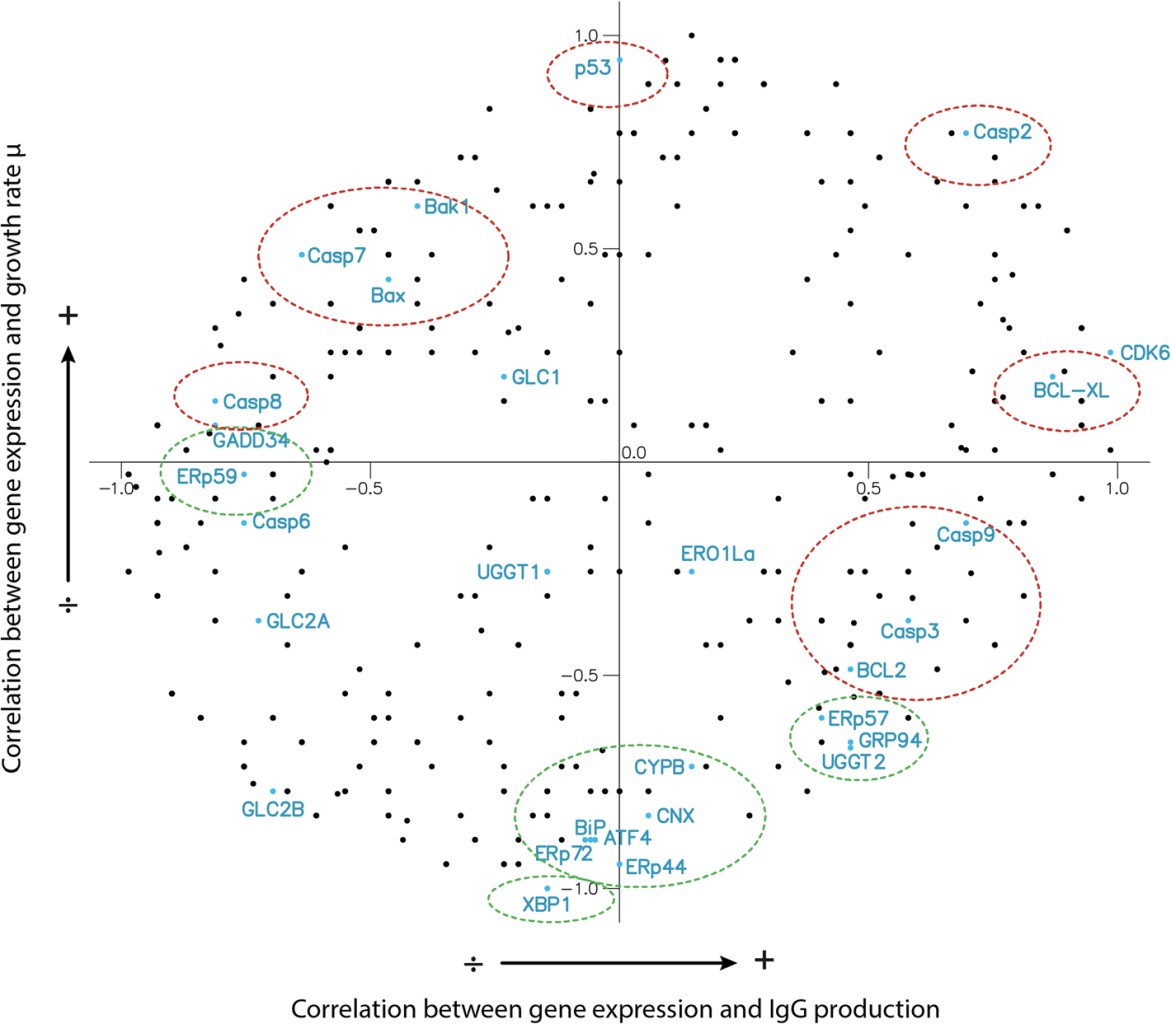
Components of the secretory network gene expression correlated with growth and protein production. The Spearman correlation coefficient is calculated for gene expression level to both growth rate and IgG production rate. Each dot marks a component of the secretory network. The highlighted points in blue are previously described generic targets. A *Green circle* indicates known targets associated with protein folding and UPR. *Red circles* indicate known targets associated with activation or inhibition of apoptosis

Of the 764 CHO secretory network components, 683 were analysed, 111 were found to correlate with growth using Spearman, and 123 using Pearson. For IgG production rate, these numbers were 102 and 183, respectively. Figure 5 shows a scatterplot of all calculated Spearman correlation coefficients. Known targets related to protein folding or UPR (green) and apoptosis or anti-apoptosis (red) are indicated. Targets previously reported to improve CHO cells growth, protein production, and survival are highlighted, and are intriguingly seen to be primarily at the outer rim of this visualisation. Of particular interest for CHO protein secretion engineering are the targets found in this outer rim, which have not been previously reported to improve cell growth and IgG production. The method seems to be a powerful tool to identify these.

#### Application of the functional network for interpretation of protein secretion in CHO cells

**Fig. 6:**
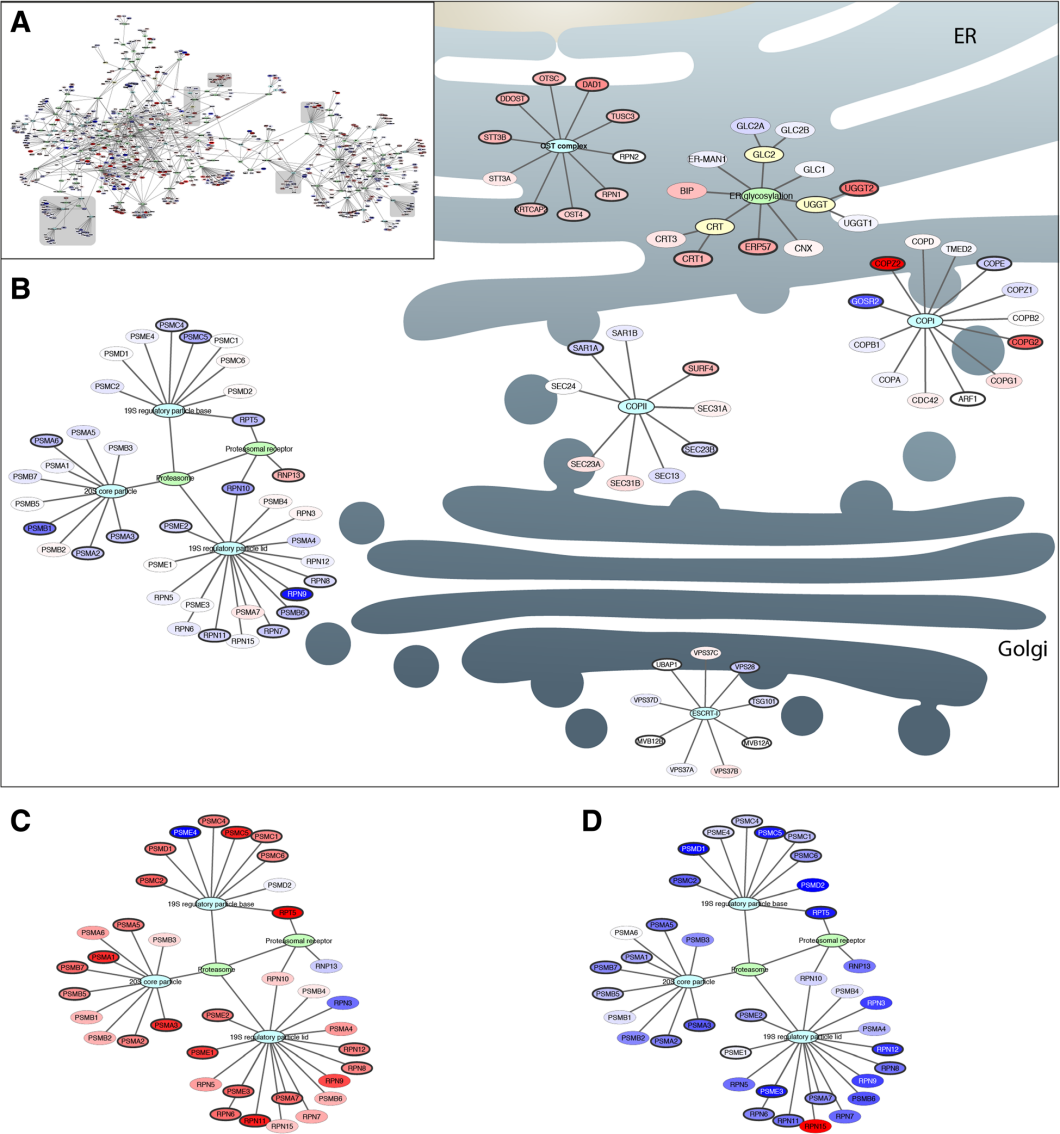
Graphical representation of the reconstructed secretory network. The change in differential expression of each components is visualised by the log2 fold change: up-regulation (*red*) and down regulation (*blue*). The intensity of the colour indicates the level in fold change. No cut-off to the fold change was added since minor changes in the expression level are important when identifying areas of activity and processes in the secretory network. Nodes are circled by a thicker line if FDR < 0.05. **a** The complete network graphically visualised in Cytoscape. **b** Selected protein complexes of OST, COPI, COPII, ESCRT-I, and the functional group of ER glycosylation and proteasome displayed in their position within the secretion pathway. **c** Proteasome components overlaid with gene expression data with the effect of secretion stress. **d** Proteasome protein/functional complex overlaid with gene expression data with the difference of exponential growth phase and stationary phase. Nodes: *Green*, Function; Turquoise, Proteins complex; *Yellow*, isoprotein; *Red*, Up-regulated; *Blue*, Down-regulated

Finally, we wanted to apply the graphical visualization of the CHO secretory RECON to facilitate interpretation of the effects related to secretion and production of a biopharmaceutical protein as IgG. The differential expression data for the effect of recombinant IgG production was visualized with the network. Despite the overall deregulation shown in Fig. 4, the network clearly visualizes e.g. that the protein complexes OST, COPI, COPII, ESCRT-I, the proteasome and the functional group of ER glycosylation are co-regulated genes of protein complexes. The subnetworks displaying these complexes are displayed in their position of the secretory pathway for illustration (Fig. 6b). Strikingly, all subunits of the proteasome are differentially expressed, but with a log fold change below 2. Therefore, examined individually, the subunits would be discarded as not significantly expressed. However, using the information of the RECON, allows us to examine all subunits as a complex, and here we see that the complex is significantly (*p* < 0.05) down-regulated in CHO cells optimised for IgG protein production. The genes of the proteasome complex are in Fig. 6c and d visualised with the gene expression values from the experiments of secretion stress and cultivation phases, respectively. Overall, the visualization of the secretory RECON allows us to identify patterns in omics-data within the secretory pathway, thus making it easier to interpret the data within the important area of protein secretion.

## Discussion

Motivated by the complexity of the secretory pathway, we have developed a network reconstruction of the secretory machinery using a systems biology approach based on manual curation. In this study, we have provided a catalogue of 801 proteins from the mouse with functional annotation and their interconnectivity. The functional annotation of the components and their grouping in subsystems were based on literature. Furthermore, we provide an implementation of this network that integrates with multi-omics data for visualization of genome-scale data. Prior work in this area includes a reconstruction of the yeast secretory machinery presented by Feizi et al. [13], which was based on well-defined stoichiometry reactions as a part of a genome-scale model reconstruction of metabolism. This study reports a network of 163 components in yeast. For the more complex organism *Aspergillus oryzae,* Lui et al. [30] presented a reconstruction of the secretory pathway using the yeast network as a base. They reported a list of 369 genes (putative end experimentally verified), including biosynthesis of GPI and dolichol. The network presented in this study covers mammalian protein secretion, excluding the N- and O-glycosylation, therefore the GPI biosynthesis and dolichol pathways are not included. The components of the cell wall, which are naturally not part of the mammalian secretory network, are also not included. The network of our study thus includes more biological processes linked to the secretion pathway than any previous study. Furthermore, we include aspects of the processes of stress in connection with heterologous protein production, specifically components of the subsystems: autophagy, apoptosis, and ER stress. The subsystems: translocation, protein folding, protein transport, UPR, and ERAD comprise a total of 512 components. Moreover, the network can be expanded and improved in the future when new components or connections are identified.

We further examined – as a test of the network – whether RNA-Seq data clustered based on biological data reflects the subsets, functional groups, and complexes of the network. As could be expected for normal, healthy cells, the components of the subsystems of ERAD, protein folding, and translocation as well as the proteasome are grouped into major clusters (Fig. 2a). One cluster contained all components associated with functions related to protein folding and translocation, while the other cluster held mainly components linked to ERAD.

The complexity of the secretory pathway was also exemplified in the close biological association between protein folding and the machinery involved in identification of terminally misfolded proteins. Furthermore, the components of the proteasome were found in two tight clusters (Fig. 2b, approximately unbiased (AU) = 86). The expression pattern of the proteasome units has similarities to the PF expression patterns of Fig. 2c, as might be expected, as both are a part of the normal growth-related functions of the cell. The bottom part of Fig. 2a holds mainly ERAD-associated components (AU > 73), however, one sub-cluster (Fig. 2d) shows a significantly different expression pattern (AU > 95). However, since all samples are from healthy growing tissues, activity of ERAD is not expected to occur, thus explaining that stress-related ERAD-associated components may not be induced in these samples.

In summary, clustering of the transcriptome data was used to assess the functional secretory network, and confirms that the literature-based sorting of the proteins into the subsystems and functional groups of ERAD, protein folding, and translocation seems meaningful. Despite the high complexity of the secretory pathway, we see that our functional categories are representative of the un-supervised clusters formed from analysis of RNA-Seq data. This also demonstrates that such analysis can provide meaningful data on the biological system by querying the network.

The functional secretory network based on the well-characterised organism mouse, as well as human and yeast, provided the foundation for constructing of a CHO cell secretory network. Despite the fact that the CHO-K1 genome is still at the draft stage, 764 homolog components were identified. For the absent 39 components, the cause is most likely either missing annotation [28] or gaps in the genome. However, wrong annotations of identified components are also likely to be present, but in a limited number since the BLAST was performed by manual curation of significant hits. As only <5% of the identified mouse network is missing, the CHO network is still a comprehensive representation of CHO protein secretion.

In order to apply the secretory RECON for studying protein secretion and to identify novel engineering targets, transcriptomic data was applied from healthy mouse tissues as reference and generated for CHO cell lines.

The transcriptome data of CHO showed that for the effect of IgG production, the high number of differentially expressed genes (6540) confirms that heterologous protein production affects the overall gene expression and general cellular processes (Table 5). This was confirmed by GO enrichment analysis, which identified biological processes terms within various types of regulation. However, using differential gene analysis alone, it was difficult to approach more specific traits within protein secretion for the IgG production.

When examining the differences due to the change of cultivation phases, few genes (333) were significantly changed more than |logFC| > 2. This is perhaps to be expected, as the experimental design removes differences between the cell lines and eliminates all growth-related genes which do not change.

Our addition of NaBu which causes hyperacetylation [8], and leads to increased transcription as well as increased recombinant protein expression [8, 14, 45], gave rise to the highest number of differentially expressed genes (8121) with a false discovery rate (FDR) < 0.05. Of these, the majority are upregulated, as to be expected with the NaBu effect of transcriptional activation. The difference between the cell lines adapted and grown in medium with or without supplementation of NEAA respectively was very little, which was confirmed by the few differentially expressed genes (27 with FDR < 0.05 and 5 having |logFC| > 2). The identified differentially expressed genes were not connected to amino acid metabolism in literature. Based on this, and the low number, we believe that they might be false positives.

We examined the transcription levels of the components from the major subsystems protein folding, translocation, proteasome, and ERAD, and observed tighter clustering in mouse than in CHO cells. This is interesting, as the mouse samples are from different tissues, while the CHO samples are the same cell type. We thus see it as a sign of less tight regulation in the cancerous CHO cells than in the healthy mouse cells. As an example, the eight subunits of the OST protein complex (addition of N-glycans on proteins in the ER lumen), cluster tightly in mouse (Fig. 2a), but have a very diverse expression profile in CHO. One exception from the apparent difference in regulation is chaperones, which are observed to have similar expression profiles in mouse CHO. Another interesting observation is that five components of the translocation complex Sec61 cluster together with protein folding components in mouse (as would be expected), while in CHO cells they clustered with components of ERAD. This could indicate that the retro-translocation function of this protein complex might be more active in CHO cells. We therefore speculate that CHO cells in general are de-regulated, at least compared to healthy mouse cells, but in traits where there has been a deliberate selection for functionality – e.g. within folding of (heterologous) proteins – the regulation has been retained.

In this study, we further presented three alternative methods to study protein secretion using omics data illustrated with transcriptomic data: Method 1) functional clustering of the secretory network for identification of regulators, Method 2) correlation of the specific IgG production and maximum growth rate to the expression levels within the secretory network, and Method 3) graphical representation of the secretory network as a method for studying protein secretion from a holistic view, and with the possibility of focusing on specific subsystems or protein complexes.

Method 1 enables identification of regulators of selected functions. Here, we identified possible regulators of protein folding, as such potential targets for cell engineering. The identified histone-binding protein (Rbbp7) (Fig. 3a) could serve as a potential target since in literature it is described as a co-repressor [15, 47]. The Methyl-CpG-binding protein (Mecp2) was identified as anti-correlating to protein folding (Fig. 3b) and could be of particular interest as a target, since it has been associated with regulating expression of a wide range of genes and that it can function as both an activator and repressor of transcription [9]. However, these two potential discoveries proved difficult to transfer to CHO, partially due to genome quality and partially due to apparent deregulation: The identified repressor/activator Mecp2 in mouse was not annotated in the CHO-K1 draft genome, and Rbbp7 was observed to have a significantly different expression profile in CHO cells (see Additional file 3: Figure S2).

For method 2 – correlating expression levels to IgG productivity and growth, we used the CHO transcriptome data for protein production. In Fig. 5 it is noticed that several of the known targets for optimised protein production in CHO cells (XBP1, ATF4, BIP, ERP72/PDI4, CNX [27, 32] have high negative correlation to growth, but interestingly with little correlation to IgG production. Possibly, many of these are a part of a stress response under normal regulation, and therefore correlate with low growth rates. In the other end of the growth axis is the gene p53 which is highly correlated with growth, but not correlated to IgG production, which is expected as it is a well-described target for improved cell viability [3]. Similar improvements are reported for the genes BAX and BAK1 [2], here they correlate only to some degree with both growth and protein production (negative correlation). In contrast, the genes P4Hb/PDI1/ERP59 and GADD34 [32] also previously described as positive targets for protein production, are located as negatively correlated to IgG production and with no correlation to growth. Within genes that correlate highly with IgG production, we see the targets known for cell survival, e.g. BCL-XL, possibly suggesting that our cells are stressed by the protein production. Other proteins previously described as positive targets for protein production are ERP57/PDIA3 [32] and Hsp90b1/GRP94 [12], but here we see that they are correlated with growth and not significantly with protein production. Finally, the different caspases are scattered across the plot and are not correlated with either growth or IgG production. This however is easily explained, as caspases are regulated by phosphorylation, which cannot be seen at the transcriptional level.

It is interesting that we see discrepancies between our calculated correlations, and approximately half of the previously reported targets. Possibly this supports that reported improvements are often cell line or in particular protein specific. However, several are identified in accordance with literature. Possibly more interesting, is how we see that known targets placed at the rim of Fig. 5, suggesting that genes placed here are interesting targets in general. In particular, novel engineering targets within the secretory pathway might be found in close proximity to the known successful targets.

Method 3 was the use of a graphical representation of the secretory RECON for studying the specific subsystems and protein complexes of protein secretion that could not be observed by simple differential gene expression analysis (too many genes) or GO enrichment analysis (too broad terms) (Fig. 6).

Illustrated by the example of the OST protein complex, all subunits of the complex, which we could identify in the CHO-K1 genome, are up-regulated (*p* = 0.2 Fisher’s exact test) in comparison with the complete network. In the same way, the majority of the expressed subunits of the proteasome protein complex were found to be down-regulated, which in comparison with the complete network is found highly significant with a *p*-value < 0.05. Importantly, none of the components has any change above 2 fold, meaning that they would not be found in a regular differential expression analysis. In contrast, the functional group of ER glycosylation components is not found to be significantly up or down-regulated. Furthermore, the subunits of the two transport complexes between the ER and the Golgi compartment, COPI and COPII, were found to have diverse expression patterns. Within a protein complex, it is expected that all the subunits have relatively similar expression patterns as observed for the mouse gene expression data (see Fig. 2). Once again, this suggests a lower or less strict level of regulation in the CHO cancer line cells.

## Conclusions

In this study, we have generated a comprehensive catalogue of characterized proteins of the secretory pathway with functional annotation and their interconnectivity and functions, and thus – to our knowledge – established to date the most elaborate RECON of the secretion pathway. The secretory network was mapped for both the well-characterised mouse (801 components) and the relatively uncharacterised CHO cell line (764 components). The RECON serves as a frame for meaningful interpretation of omics data. In particular, we present three different methods to study protein secretion through omics data: 1) Using clustering of the transcription levels of the RECON elements to identify new potential regulators. 2) Correlation of transcriptome to IgG production and growth. 3) a graphical presentation for analysing transcriptome data in relation to protein complexes or functional groups. All three are highly useful tools as demonstrated through specific findings and the general observation in several methods that CHO cells seem to have less strict transcriptional regulation than the healthy mouse cells.

The secretory pathway RECON therefore represents a strong tool in optimization of protein production and growth of CHO cell lines, the main platform for mammalian protein production.

## Additional files

Additional file 1:
**Table S1.** The full RECON. **Table S2.** The core components of the protein secretory machinery. **Table S3.** Cytoscape Input. **Table S4.** Summed Spearman correlation coefficients of the individual gene pairs of sub-cluster with the functional annotation ‘protein folding’ correlation to the complete mouse transcriptome. **Table S5.** Differential gene expression analysis of IgG production in CHO cells. **Table S6.** Differential gene expression analysis of cultivation phases in CHO cells. **Table S7.** Differential gene expression analysis of secretion stress caused by NaBu in CHO cells. **Table S8. **Differential gene expression analysis of 0% NEAA sup. in the medium for CHO cells. **Table S9.** GO enrichment analysis of CHO transcriptome data. **Table S10.** Correlation coefficients for Gene expression level correlated with protein and growth within the secretory network using RECON. (XLSX 11710 kb)
Additional file 2: Cytoscape input file. Cytoscape_functional-secretory-network. (XML 6329 kb)
Additional file 3:
**Figure S1.** Characterization of the RNA-Seq data set. **Figure S2.** Hierarchical cluster analysis with average-linkage of CHO cells expression levels. **Figure S3.** Expression profiles in CHO cells. (DOCX 751 kb)

## Abbreviations

ATCC: American Type Culture Collection
AU: Approximately unbiased
CHO: Chinese Hamster Ovary
CPM: Counts per million
ER: Endoplasmatic reticulum
ERAD: ER-associated degradation
FC: Fold change
FDR: False discovery rate
GO: Gene ontology
IgG: Immunoglobulin G
KEGG: Kyoto Encyclopedia of Genes and Genome
NaBu: Sodium butyrate
NEAA: Non-essential amino acids
ORF: Open reading frame
PBS: Phosphate-buffered saline
PF: Protein folding
PT: Protein transport
PTMs: Post-translational modifications
RECON: Reconstruction
RNA: Ribo-nucleic acid
UPR: Unfolded protein response
USA: United States of America
USD: United States Dollars
YSI: Yellow spring instruments
