# Supplementary material for: Network reconstruction of the mouse secretory pathway applied on CHO cell transcriptome data
Source: BMC Syst Biol. 2017 Mar 15;11:37. doi: 10.1186/s12918-017-0414-4 (PMC5353859; doi:10.1186/s12918-017-0414-4)
Supplement: Additional file 3: — Figure S1. Characterization of the RNA-Seq data set. Figure S2. Hierarchical cluster analysis with average-linkage of CHO cells expression levels. Figure S3. Expression profiles in CHO cells. (DOCX 751 kb) [file 12918_2017_414_MOESM3_ESM.docx]

**Additional file 3**

Figure S1 - Characterization of the RNA-Seq data set. Multi-dimensional scaling of the leading biological coefficient of variation for the RNA-Seq data set. Distances correspond to leading log-fold-changes (FC) between each pair of RNA samples. The numbering of samples corresponds to Table 2. (A) All CHO RNA-Seq samples of the dataset. (B) CHO RNA-Seq samples used for analysis the difference in gene expression between exponential growth phase and stationary phase. (C) CHO RNA-Seq samples for analysing the effect of the medium supplement NEAA. (D) CHO RNA-Seq samples used for analysing secretory stress induced by NaBu.

**
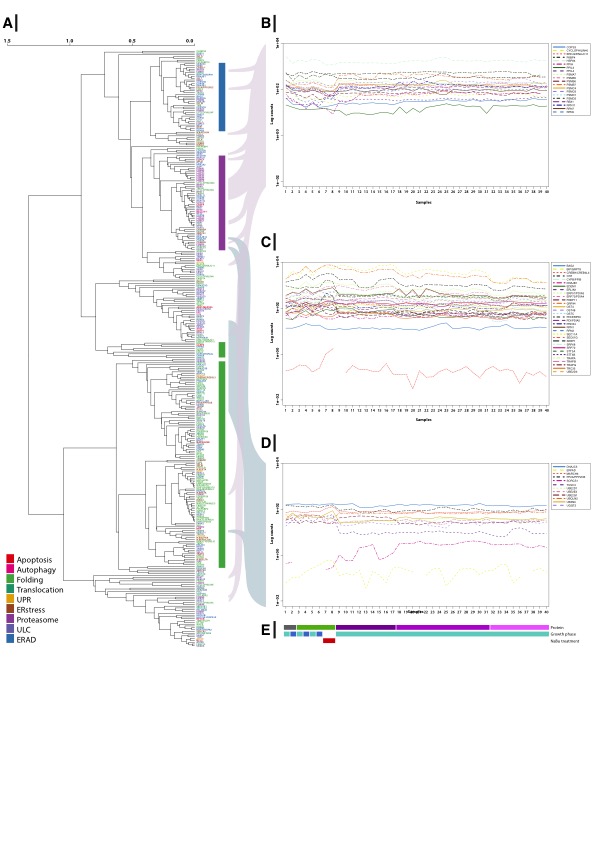
**

Figure S2 - Hierarchical cluster analysis with average-linkage of CHO cells expression levels. (A) Dendrogram representing the hierarchical clustering of the gene expression levels of the components from the subsystems of ERAD, PF, and translocation. Vertical colour bar: Purple, Proteasome; Blue, ER associated degradation (ERAD); Green, Protein folding (PF) and translocation. (B) Gene expression levels across all samples for components with the functional annotation proteasome clustering in mouse. Purple shadow indicates the components clustering in mouse location in the hierarchical cluster of CHO. (C) Gene expression levels across all samples for components with the functional annotation protein folding (PF) clustering in mouse. (D) Gene expression levels across all samples for components with functional annotation ERAD that clustered in mouse. Blue shadow indicates the components clustering in mouse location in the hierarchical cluster of CHO. (E) Identifier of samples. Top line: protein expressed; no recombinant proteins (grey), IgG (green), and FVIII high levels (dark purple), FVIII medium levels (purple), FVIII low levels (light purple). Middle line: cultivation phase; exponential growth (light blue), stationary phase (Dark blue). Bottom line: NaBu treatment (red).

Figure S3 - Expression profiles in CHO cells. Expression profile of the gene Rbbp7 (red) across all CHO samples visualised together with the expression profiles of the components of the selected cluster from Fig 2C.
